# miR-129-5p as a biomarker for pathology and cognitive decline in Alzheimer’s disease

**DOI:** 10.21203/rs.3.rs-3501125/v1

**Published:** 2023-11-01

**Authors:** Sang-Won Han, Jung-Min Pyun, Paula J Bice, David A. Bennett, Andrew J. Saykin, SangYun Kim, Young Ho Park, Kwangsik Nho

**Affiliations:** Chuncheon Sacred Heart Hospital; Soonchunhyang University Seoul Hospital; Indiana University School of Medicine; Rush University Medical Center; Indiana University School of Medicine; Seoul National University Bundang Hospital, Seoul National University College of Medicine; Seoul National University Bundang Hospital, Seoul National University College of Medicine; Indiana University School of Medicine

**Keywords:** Alzheimer’s disease, Braak, CERAD, cognition, machine learning, microRNA, miRNA-129-5p, module, network

## Abstract

**Background:**

Alzheimer’s dementia (AD) pathogenesis involves complex mechanisms, including microRNA (miRNA) dysregulation. Integrative network and machine learning analysis of miRNA can provide insights into AD pathology and prognostic/diagnostic biomarkers.

**Methods:**

We performed co-expression network analysis to identify network modules associated with AD, its neuropathology markers, and cognition using brain tissue miRNA profiles from the Religious Orders Study and Rush Memory and Aging Project (ROS/MAP) (N = 702) as a discovery dataset. We performed association analysis of hub miRNAs with AD, its neuropathology markers, and cognition. After selecting target genes of the hub miRNAs, we performed association analysis of the hub miRNAs with their target genes and then performed pathway-based enrichment analysis. For replication, we performed a consensus miRNA co-expression network analysis using the ROS/MAP dataset and an independent dataset (N = 16) from the Gene Expression Omnibus (GEO). Furthermore, we performed a machine learning approach to assess the performance of hub miRNAs for AD classification.

**Results:**

Network analysis identified a glucose metabolism pathway-enriched module (M3) as significantly associated with AD and cognition. Five hub miRNAs (miR-129-5p, miR-433, miR-1260, miR-200a, and miR-221) of M3 had significant associations with AD clinical and/or pathologic traits, with miR129-5p by far the strongest across all phenotypes. Gene-set enrichment analysis of target genes associated with their corresponding hub miRNAs identified significantly enriched biological pathways including ErbB, AMPK, MAPK, and mTOR signaling pathways. Consensus network analysis identified two AD-associated consensus network modules, and two hub miRNAs (miR-129-5p and miR-221). Machine learning analysis showed that the AD classification performance (area under the curve (AUC) = 0.807) of age, sex, and *apoE* ε4 carrier status was significantly improved by 6.3% with inclusion of five AD-associated hub miRNAs.

**Conclusions:**

Integrative network and machine learning analysis identified miRNA signatures, especially miR-129-5p, as associated with AD, its neuropathology markers, and cognition, enhancing our understanding of AD pathogenesis and leading to better performance of AD classification as potential diagnostic/prognostic biomarkers.

## Background

Alzheimer’s disease is a prevalent cause of dementia, accounting for approximately 60–80% of cases [1]. It is characterized by the extracellular deposition of amyloid-β (Aβ) in the form of diffuse and neuritic plaques (NPs) and the presence of intracellular neurofibrillary tangles (NFTs) comprised of aggregated hyperphosphorylated tau protein [2]. However, the exact mechanisms underlying the pathogenesis of Alzheimer’s disease remain unclear due to the involvement of complex neurochemical and genetic factors [3]. Dysregulated expression of microRNAs (miRNAs) is a potential mechanism contributing to gene expression changes in Alzheimer’s disease [4–6].

miRNAs are endogenous single-stranded RNA molecules approximately 20–23 nucleotides long [7]. They primarily repress the translation of specific messenger RNAs (mRNAs) by binding to their 3′-untranslated regions (3′-UTR) [8]. Several miRNAs have been identified as being dysregulated in Alzheimer’s disease, with certain miRNAs being highly expressed in the brain [4, 5, 9, 10]. However, there has been limited consensus among studies conducted on relatively small or modest sample sizes [11].

The construction of network modules based on the correlation of miRNA expression profiles can reveal the global properties of biological organization [12], given the assumption that miRNAs involved in similar functions tend to be co-expressed [13]. The weighted gene co-expression network analysis (WGCNA) approach is a method that focuses on gene co-expression networks and has been useful in describing the system-level correlation structure among transcripts [14]. Additionally, the network-based approach is a dimensionality reduction technique for analyzing high-dimensional omics data, providing insights into the pathogenesis of multifactorial disorders [15]. Therefore, the WGCNA approach has been utilized to enhance our understanding of the pathogenesis of Alzheimer’s disease [16].

In this study, using miRNA expression profiles from a large longitudinal study of aging, the Religious Orders Study and Rush Memory and Aging Project (ROS/MAP) (N = 702), as a discovery sample, we performed differential expression analysis and co-expression network analysis to identify Alzheimer’s dementia (AD)-associated network modules and their hub miRNAs. We also investigated their association with neuropathological markers [2] and cognition. After selecting target genes of the hub miRNAs, we performed association analysis of the hub miRNAs with their target genes and then differential expression analysis of target genes using brain tissue RNA-Seq data. For replication analysis, we performed a consensus miRNA co-expression network analysis to identify AD-associated consensus network modules and their hub miRNAs using the ROS/MAP dataset and an independent miRNA expression profile dataset (N = 16) from Gene Expression Omnibus (GEO). Finally, we employed a machine learning approach to assess the performance of hub miRNAs for the classification of AD.

## Methods

### Study samples

Two independent datasets were used: ROS/MAP and GEO. In the ROS/MAP cohort, subjects were categorized as having no cognitive impairment (NCI) or AD [17, 18]. In this study, to achieve a more robust differentiation between AD and NCI by employing both clinical and neuropathology criteria, AD was defined by Braak NFT scores [19] ≧ 4, Consortium to Establish a Registry for Alzheimer’s Disease (CERAD) scores [20] of definite Alzheimer’s disease (frequent NPs) or probable Alzheimer’s disease (moderate NPs), and cognitive diagnosis of probable Alzheimer’s disease with no other causes. NCI was defined by Braak scores ≦ 3, the CERAD scores of possible Alzheimer’s disease (sparse NPs) or no Alzheimer’s disease, and clinical diagnosis of no cognitive impairment [21]. In the GEO (GSE157239) dataset from the Human Brain Bank of the Brazilian Aging Brain Study Group, AD was defined by Braak NFT scores [19] ≧ 3 and NCI as subjects without neuropathological lesions or neurological signs, as previously described [22].

### miRNA profiling data

For the ROS/MAP cohort, miRNA profile data were downloaded from the Accelerating Medicines Partnership for Alzheimer’s Disease (AMP-AD) Knowledge Portal on Synapse (syn3387325) (www.synapse.org). The miRNAs were extracted from brain tissue in the dorsolateral prefrontal cortex (DLPFC) using the Nanostring nCounter Human miRNA Expression assay kit and annotated using definitions from the miRBase [23]. These miRNAs were eluted from the miRNeasy spin columns in buffer and tested by Nanodrop and Bioanalyzer RNA 6000 Nano Agilent chips. The dataset consisted of 309 miRNAs from 702 individuals after correcting for the probe-specific backgrounds and performing a three-step filtering of sample and miRNA expression [6, 24]. The miRNA data were normalized using a combination of quantile normalization and Combat [25] to remove batch effects. The miRNAs identified by microarray were validated with specific real-time reverse transcription PCR (qRT-PCR) assays, as previously described in detail [6, 24].

For the GEO dataset, miRNA profiles were downloaded from the National Center for Biotechnology Information as accession numbers GSE157239 (www.ncbi.nlm.nih.gov/projects/geo/). The miRNAs were extracted from brain tissue in the temporal cortex, profiled using microarray in the Affymetrix miRNA Array, and annotated using definitions from the miRBase [23]. After the isolation and biotin labeling of the miRNAs was performed, the labeled miRNAs were hybridized to the GeneTitan instrument with the Array Strip Hybridization kit [26]. Quality control was performed using the Expression Console software [27], and results were exported for processing in the Transcriptome Analysis Console software [28]. The miRNA identified by microarray were also validated via qRT-PCR, as previously described [22].

### RNA-Sequencing for mRNA expression data in the ROS/MAP

RNA-Seq data generated from brain tissue in the DLPFC were downloaded from the AMP-AD Knowledge Portal on Synapse (syn8456638) (www.synapse.org). The sequencing was performed on the Illumina HiSeq2000 with 101 base pair paired-end reads, targeting a coverage of 50 million paired reads, as detailed in previous studies [24, 29]. The reads were aligned to the human genome reference (hg19) using Tophat [30] with Bowtie1 as the aligner [31]. The expression levels of transcripts were estimated using the Gencode V14 annotation with the RSEM package [32]. FPKM (Fragments Per Kilobase of transcript per Million mapped reads) normalization was applied to the mRNA expression data. The log2 counts-per-million (logCPM) values generated in 634 subjects were finally used for further analysis, as previously described [33].

### Assessment of CERAD, Braak, and cognition in ROS/MAP

Definite or probable Alzheimer’s disease relative to possible or no Alzheimer’s disease was based on CERAD, which is a semiquantitative measure of neuritic plaques, as previously reported [20, 34, 35]. Braak scores [19] ≧ 4 relative to ≦ 3 were used to dichotomize neurofibrillary tangles as previously reported [34, 35]. We used the term ‘CERAD positive’ for ‘probable’ or ‘definite’ Alzheimer’s disease, and ‘CERAD negative’ for ‘possible’ or ‘no’ Alzheimer’s disease based on CERAD. We also used the term ‘Braak positive’ for Braak scores ≧ 4, and ‘Braak negative’ for Braak scores ≦ 3. Cognitive performance was assessed longitudinally by Z-scores of global cognitive performance averaged across 19 tests spanning five cognitive domains, including episodic memory, working memory, semantic memory, perceptual speed and visuospatial ability/perceptual orientation, as previously reported [36].

### Co-expression miRNA network construction

A scale-free miRNA co-expression network was constructed using the WGCNA package based on miRNA expression profiles [14]. The “pickSoftThreshold” function was used to select an appropriate soft threshold power β that achieves a scale-free topology, and the network adjacency matrix was calculated based on co-expression similarity. Unsupervised hierarchical clustering with the dynamic tree cut procedure was used to identify modules of co-expressed miRNAs. Each module was represented by a module eigengene (ME), which was defined as the first principal component of the expression matrix representing the overall level of miRNA expression within each module.

### Identification of hub miRNAs in an AD-associated network module and their target genes

The “softConnectivity” function was used to identify the top 10 hub miRNAs with the highest intramodular connectivity according to the Topological Overlap Matrix (TOM) based on intramodular connectivity measures. The target genes of these hub miRNAs were predicted using the TargetScan [37] and the miRDB [38] databases, which provide both predicted and experimentally verified interaction information between miRNAs and genes. The overlapping target genes between the two databases were selected for further analysis.

### Association of AD-associated hub miRNAs with their target genes using RNA-Seq data in the ROS/MAP

After target genes of AD-associated hub miRNAs were selected, brain tissue-based RNA-Seq data analyses were performed to assess whether the predicted target genes were associated with expression levels of their corresponding miRNAs.

### Pathway-based enrichment analysis of target genes and miRNAs

Gene-set enrichment analysis was performed using the Database for Annotation, Visualization and Integrated Discovery (DAVID) online tool [39] to identify the biological pathways of target genes significantly associated with the expression levels of AD-associated hub miRNAs. The Kyoto Encyclopedia of Genes and Genomes (KEGG) [40] and Gene ontology (GO)-biological processes (BP) [41] pathways were used.

miRNA-set enrichment analysis was also performed using the TAM 2.0 [42] tool to characterize the functional annotations of the miRNA set within each module through overrepresentation analysis. miRNA-set enrichment analysis was restricted to pathways containing 3 or more miRNAs. The most strongly associated biological processes for the involvement of the central nervous system were selected for each module. Bonferroni correction [43] was applied to adjust for multiple testing.

### Consensus network construction

A consensus miRNA co-expression network was constructed across the ROS/MAP and GEO (GSE46579) datasets for replication using the WGCNA package [14]. The “blockwiseConsensusModules” function was used to construct modules in the consensus network based on pairwise miRNA dissimilarity measures. The importance of a miRNA in a network module was determined by kME, defined as the strength of the correlation of expression levels of a miRNA with the ME. Hub miRNAs in a consensus module were defined as those miRNAs with an absolute kME value greater than 0.7.

The “modulePreservation” function was used to construct a preservation network based on the correlation between all pairs of consensus ME values across the two networks from the ROS/MAP and GEO datasets. A density (D) of the eigengene network, defined as the average scaled connectivity, was estimated to discover changes in preservation patterns across each consensus module. The D value close to 1 indicates strong preservation of the correlation patterns between all pairs of eigengenes across the two networks [44, 45].

### Statistical analysis

For differential expression analysis, logistic regression models were used to investigate the association of miRNAs expression levels with AD, CERAD, and Braak stage. For co-expression miRNA network analysis, logistic regression models were used to evaluate the association of ME values of network modules with AD and to identify AD-associated network modules. Linear regression models were used to perform the association analysis of miRNA expression levels and ME values of network modules with global cognitive performance at the last visit, while linear mixed effects models were used to investigate their association with longitudinal changes of global cognitive performance.

For differential expression analysis using RNA-Seq data of the target genes identified as significantly associated with expression levels of AD-associated hub miRNAs, logistic regression models were used to investigate the association between expression levels of the target genes and AD.

Covariates in the association analysis with AD and neuropathological markers included age, sex, study (ROS or MAP), RNA integrity numbers, and post-mortem interval. For the association analysis with cognitive performance, education was included along with the aforementioned covariates. The false discovery rate (FDR) correction was used to adjust for multiple testing with the Benjamini-Hochberg procedure [46] unless otherwise specified.

The STREAMLINE tool [47], a machine learning approach using penalized logistic regression, was used to investigate the classification performance of AD-associated hub miRNAs in differentiating AD from NCI. This approach was chosen to reduce the effect of multicollinearity on feature selection [48]. The data were randomly divided into 70% used for training the model and 30% used for testing, with a 10-fold cross-validation procedure. The classification performance of five different models was evaluated using the receiver operating characteristic (ROC) curve and the area under the receiver operating characteristic curve (AUC). Training features in Model 1 included age, sex and apolipoprotein E (*APOE*) ε4 carrier status; training features in Model 2 included five AD-associated hub miRNAs; training features in Model 3 included age, sex, APOE ε4 carrier status and five AD-associated hub miRNAs; training features in Model 4 included all 309 miRNAs, and training features in Model 5 included age, sex, APOE ε4 carrier status and all 309 miRNAs. Paired t-tests were performed to compare the AUC results for the different models [49].

**Figures 1:**
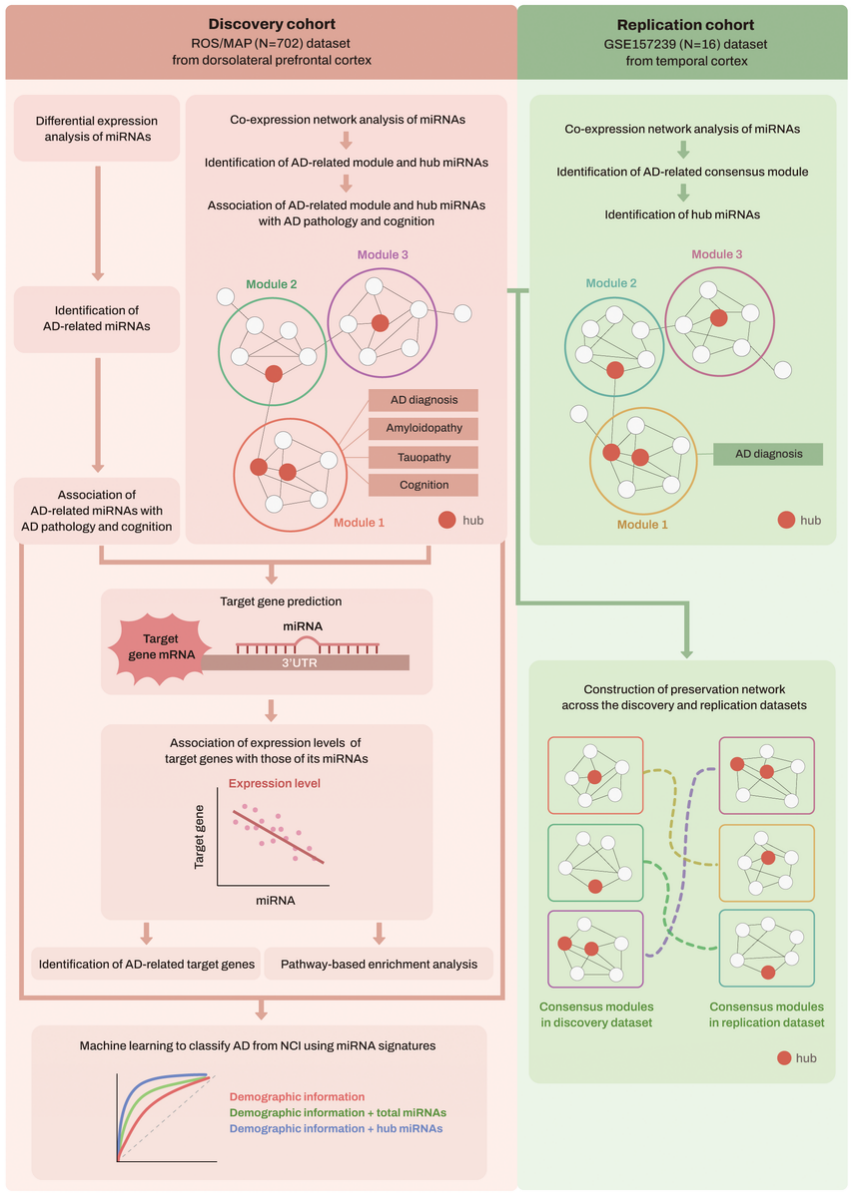
Schematic overview of the workflow of our analysis Abbreviations: AD, Alzheimer’s dementia; MAP, Memory and Aging Project; miRNAs, microRNAs; mRNAs, messenger RNAs; NCI, no cognitive impairment; ROS, Religious Orders Study; 3′-UTR, 3′-untranslated region.

All statistical analyses were conducted using R version 4.2.0, and statistical significance was set at a *P*-value of 0.05 after adjusting for multiple comparisons. Figure 1 shows a workflow of all analysis steps used in this study.

## Results

The ROS/MAP cohorts with miRNA consisted of 702 participants, including 102 NCI and 177 AD, with a median age of 88.5 at death and 64.1% females. The GEO (GSE157239) dataset consisted of 16 participants, including 8 NCI and 8 AD, having miRNA data, with a median age of 81.5 at death and 68.8% females. Table 1 and **Table S1** show the demographic characteristics of these individuals.

### Differential expression analysis of miRNAs

**Figure 2:**
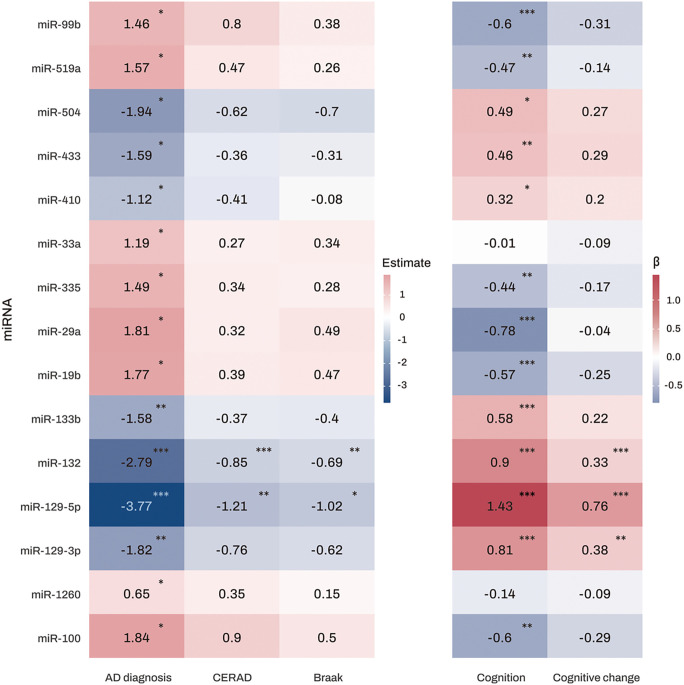
Heatmap of association analysis results of miRNAs with diagnosis and clinical and pathological traits The numbers represent the logistic and linear regression coefficients of association between miRNAs and traits. Stars indicate significant associations with FDR-corrected *p* value < 0.05. Red colors represent positive correlations, while blue colors denote negative correlations. The darker the color, the stronger the association. Abbreviations: AD, Alzheimer’s dementia; CERAD, Consortium to Establish a Registry for Alzheimer’s Disease; FDR, false discovery rate; miRNAs, microRNAs. Note: significance stars indicating the *p*values of the correlations adjusted for multiple comparisons * *P*-value < 0.05. ** *P*-value < 0.01. *** *P*-value < 0.001.

Differential expression analysis was performed on 309 miRNAs in 177 AD and 102 NCI subjects from ROS/MAP. 15 miRNAs were found to be significantly associated with AD (Fig. 2). Notably, expression levels of miR-129-5p and miR-132 were significantly lower in both the CERAD and Braak positive groups (Fig. 2 and **Table S2**). Additionally, higher expression levels of miR-129-3p, miR-129-5p, miR-132, miR-133b, miR-410, miR-433, and miR-504, as well as lower expression levels of miR-100, miR-19b, miR-29a, miR-335, miR-519a, and miR-99b, were significantly associated with better global cognitive performance at the last visit. Furthermore, higher expression levels of miR-129-3p, miR-129-5p, and miR-132 showed significant associations with slower longitudinal decline of global cognition.

### Co-expression miRNA network analysis

#### Modules associated with AD, CERAD, Braak, and cognition

**Figure 3:**
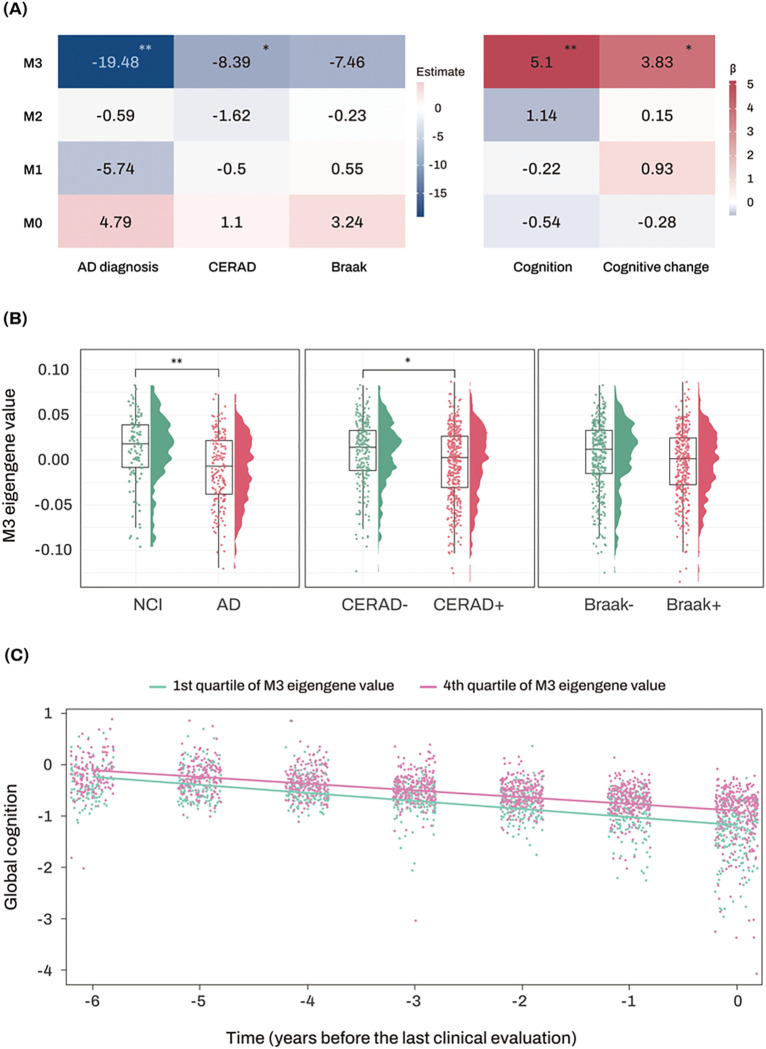
Association analysis results of module eigengenes with diagnosis and clinical and pathological traits. (A) The numbers represent the logistic and linear regression coefficients of association between module eigengenes and traits. Stars indicate significant associations with FDR-corrected *p*value < 0.05. Red colors represent positive correlations, while blue colors denote negative correlations. The darker the color, the stronger the association. (B) Violin and box plots represent the M3 module eigengene values in the diagnosis, amyloidopathy and tauopathy groups from the ROS/MAP cohort. (C) The x-axis represents years before the last clinical evaluation, while the y-axis indicates global cognitive Z scores. The two lines represent the different slopes of global cognitive changes of subjects with 1st quartile (green color) versus 4th quartile (red color) stratified by ME values of AD-associated M3 module. Abbreviations: AD, Alzheimer’s dementia; CERAD, Consortium to Establish a Registry for Alzheimer’s Disease; FDR, false discovery rate; MAP, Memory and Aging Project; ME, module eigengene; NCI, no cognitive impairment; ROS, Religious Orders Study. Note: significance stars indicating the *p*values of the correlations adjustment for multiple comparisons * *P*-value < 0.05. ** *P*-value < 0.01.

A scale-free co-expression network was constructed based on the miRNA expression profiles of 702 subjects using WGCNA. A cutting tree height of 11 was selected to eliminate outliers, and 663 individuals under the cutting tree height were kept for further analysis. A soft thresholding power value of β = 4 was selected, and four network modules were identified (**Figure S1**). The M0 module consisted of miRNAs not assigned to any other modules and was excluded in further analysis. Lower ME values of the M3 module were significantly associated with a greater likelihood of AD and CERAD, but not with Braak stage. Moreover, lower ME values of the M3 module were associated with lower global cognitive performance at the last visit and faster longitudinal decline of global cognition (Fig. 3 and **Table S3**). Enrichment analysis of miRNAs revealed that M3 was highly associated with glucose metabolism, while M1 and M2 were strongly linked to innate immunity and DNA damage response, respectively (**Table S4**).

#### Hub miRNAs associated with AD, CERAD, Braak, and cognition

**Figure 4:**
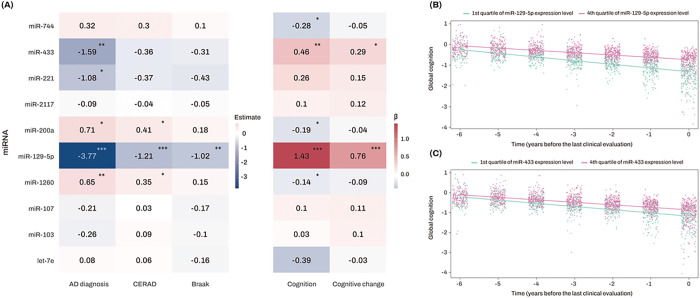
Association analysis results of top 10 candidate hub miRNAs with diagnosis and clinical and pathological traits. (A) The numbers represent the logistic and linear regression coefficients of association between miRNAs and traits. Stars indicate significant associations with FDR-corrected *p* value < 0.05. Red colors represent positive correlations, while blue colors denote negative correlations. The darker the color, the stronger the association. (B, C) The x-axis represents years before the last clinical evaluation, while the y-axis indicates global cognitive Z scores. The two lines represent the different slopes of global cognitive changes of subjects with 1st quartile (green color) versus 4th quartile (red color) stratified by expression levels of miR-129-5p (B) and miR-433 (C), respectively. Abbreviations: AD, Alzheimer’s dementia; CERAD, Consortium to Establish a Registry for Alzheimer’s Disease; FDR, false discovery rate; miRNAs, microRNAs. Note: significance stars indicating the *p*values of the correlations adjustment for multiple comparisons * *P*-value < 0.05. ** *P*-value < 0.01. *** *P*-value < 0.001.

In the AD-associated glucose metabolism pathway-enriched M3 module, the top 10 miRNAs with the highest TOM-based intramodular connectivity were identified as hub miRNAs (Fig. 4). Among these 10 miRNAs, the strongest finding was for miR-129-5p, which was inversely related to AD, CERAD, and Braak and directly related to level of and change in cognition. Higher expression levels of miR-433 and miR-221 were inversely associated with the likelihood of AD, while only miR-433 was associated with better cognition and slower cognitive decline (Fig. 4 and **Table S5**). By contrast, higher expression levels of miR-200a and miR-1260 were related to a greater likelihood of AD, higher CERAD and lower cognition, but not with Braak or cognitive decline. Finally, miR-744 was only associated with lower global cognition.

#### Target genes associated with AD for AD-associated hub miRNAs

Target genes for the five AD-associated hub miRNAs (miR-129-5p, miR-433, miR-1260, miR-200a, and miR-221) were obtained from the miRDB and TargetScan databases, resulting in 1417, 614, 567, 1526, and 1164 genes from the miRDB database, and 732, 1391, 5239, 4128, and 5878 genes from the TargetScan database. 406, 198, 512, 892, and 769 overlapping target genes for these five miRNAs, respectively, between the two databases were then identified.

RNA-Seq data analysis identified 189, 101, 310, 591, and 73 target genes as significantly associated with expression levels of corresponding miRNAs for miR-129-5p, miR-433, miR-1260, miR-200a, and miR-221, respectively. Furthermore, RNA-Seq data analysis identified a total of 22, 3, 12, 30, and 25 target genes as significantly associated with AD for miR-129-5p, miR-433, miR-1260, miR-200a, and miR-221, respectively (Table 2).

#### Pathway-based enrichment analysis of target genes

The KEGG pathway-based enrichment analysis revealed that the target genes identified through RNA-Seq analysis for the five AD-associated hub miRNAs were mainly involved in the following pathways: axon guidance, erythroblastic leukemia viral oncogene homolog (ErbB) signaling pathway, mitogen-activated protein kinase (MAPK) signaling pathway, gamma-aminobutyric acid-ergic (GABAergic) synapse, autophagy, 5′ adenosine monophosphate-activated protein kinase (AMPK) signaling pathway, mammalian target of rapamycin (mTOR) signaling pathway, and glutamatergic synapse (Table 3). The GO-BP pathway-based enrichment analysis showed that target genes identified through RNA-Seq analysis for the five AD-associated hub miRNAs were enriched in the following pathways: protein phosphorylation, nervous system development, chromatin organization, and neuron migration (**Table S6**).

#### Consensus network analysis using miRNA profiles from two independent datasets

Consensus modules and a preservation network were constructed to understand changes in preservation patterns across consensus modules. Additionally, hub miRNAs in consensus modules were selected to assess the replication of the five AD-associated hub miRNAs, identified in the ROS/MAP dataset, within an independent dataset.

#### Identification of consensus network modules

The construction of miRNA co-expression network modules was performed separately for the discovery and replication datasets, and the consensus modules were identified using the consensus dissimilarity measures in the average linkage hierarchical clustering method. A soft thresholding power value of β = 4 was selected for each dataset, and four consensus modules were identified (**Figure S2**). All consensus modules had counterparts in both datasets, indicating that the consensus module structures in two datasets were similar (**Figure S3**). The consensus CM0 module consisted of miRNAs not assigned to any other modules.

#### Preservation of consensus modules

**Figure 5:**
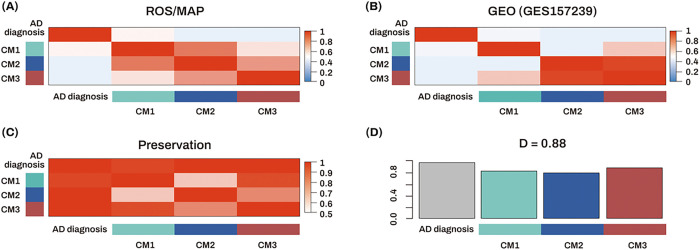
Construction of consensus module eigengene networks (A, B) Heatmaps showing the adjacencies of eigengene for each of the eigengene networks (A, ROS/MAP cohort; B, GSE157239 cohort). Each row and column corresponds to an eigengene labeled by the consensus module or diagnosis. Red colors represent positive correlations, while blue colors denote negative correlations. The darker the color, the stronger the association. (C) Adjacency heatmap displaying the pairwise preservation between the two consensus eigengene networks. Each row and column corresponds to an eigengene labeled by the consensus module or diagnosis. Red colors represent adjacency. The darker the color, the higher the adjacency. (D) Bar plot showing the preservation of correlation of consensus module eigengene between the two networks. Each colored bar corresponds to the eigengene of the associated consensus module. The height of the bar (y-axis) represents the eigengene preservation measure. The D value indicates the mean preservation of eigengene networks across the datasets. The high density value of D (= 0.88) denotes high overall preservation between the two networks. Abbreviations: AD, Alzheimer’s dementia; CM, consensus module; M, module; MAP, Memory and Aging Project; ROS, Religious Orders Study.

A consensus ME network was constructed to investigate whether expression patterns of modules were correlated with each other (Fig. 5A, B). The preservation networks of the correlations of the consensus ME pairs between the discovery and replication datasets were further constructed to understand the changes in preservation patterns of two datasets (Fig. 5C). The D value of the preservation networks between all pairs of the consensus ME across the two networks was 0.88 (Fig. 5D), indicating that these modules were well preserved in their expression patterns across the two independent datasets.

#### Identification of AD-associated consensus modules across two independent datasets and replication for AD-associated hub miRNAs

**Figure 6:**
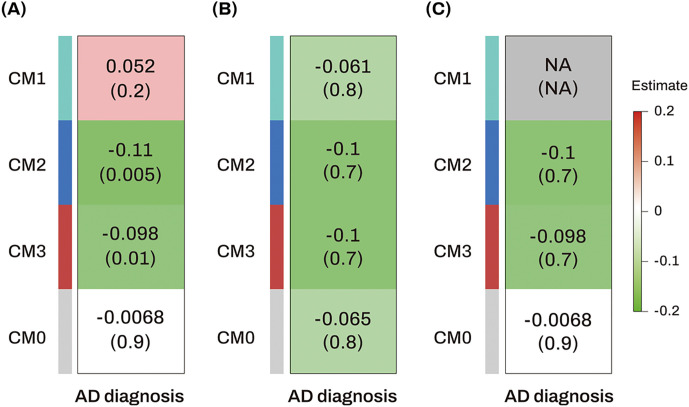
Association analysis results of consensus module eigengenes with diagnosis in the ROS/MAP cohort, GEO (GSE157239) cohort, and across ROS/MAP and GEO datasets (A, B) Heatmaps showing module-trait relationships in the ROS/MAP (A) and GEO (GSE157239) (B) cohorts. (C) Heatmaps showing consensus module-trait relationships across the ROS/MAP and GSE157239 cohorts. The numbers in the table indicate the coefficients (top) and its *p* value (bottom) of association between consensus module eigengenes and diagnosis. Red colors represent positive correlations, while green colors denote negative correlations. The darker the color, the stronger the association. Missing (NA) entry indicates the failure of forming a consensus because the directions of correlations in the ROS/MAP and GSE157239 datasets are opposite. Abbreviations: AD, Alzheimer’s dementia; CM, consensus module; GEO, Gene Expression Omnibus; M, module; MAP, Memory and Aging Project; NA, Not applicable; ROS, Religious Orders Study.

Figure 6 shows the module-clinical trait heatmaps, indicating the associations between AD and four consensus modules across two independent datasets. Lower ME values of the consensus CM2 and CM3 modules were significantly associated with AD in the ROS/MAP dataset. In the GEO dataset, although the association between ME values of the consensus CM2 and CM3 modules and AD was not significant, the effect sizes and association directions were consistent with those in the ROS/MAP dataset. Among the five AD-associated hub miRNAs identified in the discovery dataset (ROS/MAP), miR-129-5p, miR-221 and miR-200a were included in the CM2 module (**Table S7**), but none of the miRNAs were present in the CM3 module (**Table S8**). Notably, miR-129-5p and miR-221, identified as AD-associated hub miRNAs in the ROS/MAP cohort, were also hub miRNAs in an independent replication dataset (GEO) because their kME values in the replication dataset (GEO) were higher than 0.7 (Table 4 and **Table S8**). The correlation analysis showed that the M3 module from the ROS/MAP dataset and the consensus CM2 module from the combined ROS/MAP and GEO datasets were strongly correlated (correlation coefficient = 0.88) (**Figure S4**). Enrichment analysis of miRNAs identified glucose metabolism as a significantly enriched biological pathway in both M3 and CM2 (**Table S9**).

#### Machine learning analysis for AD classification

**Figure 7:**
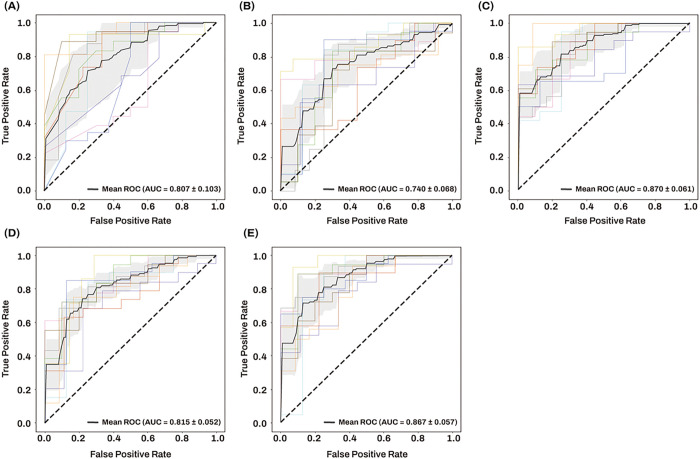
The ROC curves and mean AUC of machine learning approach using penalized logistic regression Sensitivity is on the y-axis and 1-specificity is on the x-axis. 10-fold cross validation was used to investigate and compare the classification performance of five different classification models for differentiating AD from NCI. The gray zone around the mean ROC curve represents ± 1 standard deviation. Five different classification models are shown per following training features. (A) training features include age, sex and apolipoprotein E *(APOE)* ε4 carrier status (B) training features include five AD-associated hub miRNAs (C) training features include age, sex, APOE ε4 carrier status and five AD-associated hub miRNAs (D) training features include all 309 miRNAs (E) training features include age, sex, *APOE* ε4 carrier status and all 309 miRNAs. Abbreviations: AD, Alzheimer’s dementia; AUC, area under the curve; miRNAs, microRNAs, NCI, no cognitive impairment; ROC, receiver operating characteristic.

A machine learning approach using penalized logistic regression for the classification of AD from NCI was used to evaluate five different classification models (Table 5). The results of 10-fold cross-validation are presented in Fig. 7. Model 1, including age, sex, and *APOE* ε4 carrier status, achieved a mean AUC value of 0.807 with a standard deviation of 0.103 (Fig. 7A). The mean AUC value of Model 3, obtained by adding five AD-associated hub miRNAs to Model 1, significantly increased to 0.870 with a standard deviation of 0.061 (*P* value = 0.022) (Fig. 7C), which was comparable to that of Model 5, obtained by adding all 309 miRNAs to Model 1 (Fig. 7E). The mean AUC values of Model 2, including only five AD-associated hub miRNAs, and Model 4, including all 309 miRNAs, were 0.740 and 0.815 with a standard deviation of 0.068 and 0.052, respectively (Fig. 7B, D).

## Discussion

In this study, we performed a network-based analysis of miRNAs and identified a network module (glucose metabolism pathway-enriched M3) that showed significant associations with AD, level of and change in cognition, and CERAD and Braak pathologic traits of Alzheimer’s disease. In the AD-associated glucose metabolism pathway-enriched M3 module, we identified five hub miRNAs (miR-129-5p, miR-433, miR-1260, miR-200a, and miR-221) as significantly associated with AD, neuropathologic markers, and/or cognition, with miR-129-5p being the strongest and associated with all AD traits. Gene-set enrichment analysis of the target genes of these five AD-associated hub miRNAs revealed enrichment of ErbB, MAPK, AMPK, and mTOR signaling pathways. In the replication analysis using an independent additional dataset, we identified AD-associated CM2 and CM3 modules. Remarkably, miR-129-5p and miR-221, identified as AD-associated hub miRNAs in the discovery cohort, were also found to be hub miRNAs in the replication cohort. Preservation analysis showed consistent expression patterns of the consensus modules across two independent datasets.

This study identified AD-associated miRNAs through network-based analysis, not detected in the miRNA-based differential expression analysis. Moreover, our findings shed light on the association between AD-associated miRNAs and longitudinal changes of cognition.

Among five AD-associated hub miRNAs, we found that greater miR-129-5p was associated with a lower likelihood of AD, better cognition, slower cognition decline, and lower CERAD and Braak pathologic traits of Alzheimer’s disease [6, 50–53]. miR-129-5p has been suggested to play roles in Alzheimer’s disease, potentially involving the regulation of autophagy [51], neuroinflammation [52, 54, 55], and neuronal cell death [53–55] through targeting amyloid precursor protein (APP) [51], high-mobility group box 1 (HMGB1) [55], and yes-associated protein 1 (YAP1) [53] genes. For miR-221, we observed decreased expression levels in AD but did not find any associations with neuropathological markers or cognition, which aligns with the study by Manzine et. al. [56]. For miR-200a, we observed increased expression levels in AD, consistent with previous studies [50, 57–61], highlighting its role in the Aβ-induced neuronal apoptosis and cell cycle deregulation [58–61]. Interestingly, we also identified a novel association between higher miR-200a expression levels and worse cognition. For miR-433, we observed decreased expression levels in AD and its association with cognition but not neuropathological markers, which aligns with the study by Wang et al. [62]. Lastly, miR-1260 exhibited increased expression levels in AD, consistent with previous studies [50–61]. Notably, increased expression levels of miR-1260 were associated with CERAD pathologic traits and worse cognition, which is another novel finding from our study.

Our pathway-based enrichment analysis revealed that the AD-associated predicted target genes for the five AD-associated hub miRNAs were related to ErbB, AMPK, MAPK, and mTOR signaling pathways. ErbB, involved in various biological processes, such as myelination, neurite outgrowth, cell proliferation, differentiation and protection against apoptosis, is downregulated in Alzheimer’s disease due to Aβ-mediated neurotoxicity [63–65]. AMPK, which regulates cell polarity, apoptosis, cell migration and synaptic plasticity, is activated in Alzheimer’s disease, contributing to tauopathy, synaptotoxicity and dendritic deficits [66–69]. AMPK, which regulates cell polarity, apoptosis, cell migration and synaptic plasticity, is activated in Alzheimer’s disease, contributing to tauopathy, synaptotoxicity and dendritic deficits [70–72]. Lastly, mTOR, a conserved serine/threonine protein kinase, is dysregulated in Alzheimer’s disease through Aβ-induced autophagy impairment, endoplasmic reticulum stress, cell apoptosis and mitochondrial dysfunction [73–76].

The machine learning analysis for classification of AD from NCI showed that five AD-associated miRNAs significantly improved the performance of demographic information and *APOE* ε4 carrier status for classification of AD from a mean AUC value of 0.847 to that of 0.889.

miRNAs have emerged as promising therapeutic targets in Alzheimer’s disease due to their crucial role in regulating the expression levels of target genes involved in Alzheimer’s disease pathogenesis [10, 77, 78]. Currently, two types of miRNA targets, miRNA mimics and anti-miRNAs, are being explored for therapeutic interventions in Alzheimer’s disease [10, 77].

Our study has several limitations. Firstly, the limited sample size of the replication cohort might have contributed to the decreased statistical power for replication analysis. Further studies with larger samples are needed to validate our findings. Secondly, the replication dataset lacks neuropathological markers and cognitive information, limiting our investigation for associations with neuropathology and cognition to the ROS/MAP cohort. Thirdly, miRNA expression profiles were generated using different brain regions and different microarray platforms in the discovery and replication cohorts, which may have introduced variability in the results. Lastly, the difference in the definitions of the diagnostic groups (NCI and AD) in the two datasets may have led to a potential confounding factor in the consensus network analysis.

## Conclusions

In summary, our network-based approach identified AD pathology and cognition-associated miRNAs. Notably, miR-129-5p and miR-221 were replicated in an independent dataset. The inclusion of AD-associated miRNAs improved the classification performance of AD from NCI. This integrative network approach can provide insight into AD pathogenesis and highlights these miRNAs as diagnostic/prognostic biomarkers and potential therapeutic targets for AD. However, further investigations are necessary to elucidate the underlying mechanisms and validate these findings.

## Figures and Tables

**Table 1:** Demographic information of participants from the ROS/MAP cohort

	NCI (n = 102)	AD (n = 177)	Total (n = 702)	*P* value ^b^

**Female**	54 (52.9)	126 (71.2)	449 (64.1)	0.002

**Age at death in years ^a^**	85.0 (78.8–89.0)	90.0 (87.4–90.0)	88.5 (84.1–90.0)	<0.001

**Study**				0.926
**ROS**	53 (52.0)	93 (52.5)	381 (54.4)	
**MAP**	49 (48.0)	84 (47.5)	320 (45.6)	

**Global cognitive Z scores at the last visit ^a^**	0.10 (−0.25–0.36)	−0.35 (−0.93–0.06)	−0.17 (−0.56–0.19)	<0.001

**Slope of global cognitive Z scores ^a^**	−0.03 (−0.13 – 0.02)	−0.17 (−0.30 – −0.08)	−0.09 (−0.22 – −0.01)	<0.001

**RNA integrity numbers ^a^**	7.3 (6.0–8.0)	6.7 (5.4–7.4)	6.9 (5.6–7.7)	0.001

**Post-mortem interval in hours ^a^**	6.3 (4.8–8.6)	5.8 (4.2–8.3)	5.8 (4.3–8.5)	0.124

Values are n (%), unless indicated otherwise.

Among a total of 702 participants with miRNA data, 177 subjects met criteria for AD and 102 met criteria for NCI based on neuropathological and clinical data.

a Data are presented as median (interquartile range)

b The Mann-Whitney U test or chi-square test was used to determine the *P* value for comparisons between AD and NCI groups, as appropriate.

Abbreviations: AD, Alzheimer’s dementia; MAP, Memory and Aging Project; miRNAs, microRNAs; NCI, no cognitive impairment; ROS, Religious Orders Study.

**Table 2:** Results of differential expression analysis of AD-associated target genes of five AD-associated hub miRNAs between NCI and AD. Here we showed only significantly differentially expressed target genes in AD.

miR-129–5p		miR-433		miR-1260		miR-200a		miR-221	
Target genes	*P* value^a^	Target genes	*P* value^a^	Target genes	*P* value^a^	Target genes	*P* value^a^	Target genes	*P* value^a^
*PIEZO2*	0.001	*BRWD1*	0.036	*SAMD4A*	0.005	*SLC6A9*	< 0.001	*DYNC1LI2*	0.002
*NRN1*	0.015	*NSMCE1*	0.036	*NRN1*	0.022	*C17orf58*	0.005	*MLIP*	0.012
*ZNF704*	0.015	*ST8SIA4*	0.036	*MID1IP1*	0.022	*SLFN5*	0.025	*BRWD1*	0.012
*COX15*	0.030			*LGMN*	0.023	*TNS3*	0.025	*NRIP2*	0.012
*CHD7*	0.030			*TBC1D24*	0.023	*SLC38A2*	0.025	*ZFR2*	0.012
*PCDH8*	0.030			*LRRC73*	0.033	*RUFY2*	0.027	*ZNF652*	0.012
*TOMM40*	0.030			*GOLGA8B*	0.033	*CHP1*	0.035	*EML6*	0.012
*SMARCC1*	0.030			*CCDC113*	0.036	*BRWD1*	0.035	*LPAR1*	0.012
*RAB3B*	0.030			*RHNO1*	0.041	*LRRC8B*	0.035	*KIF1C*	0.012
*TMEM67*	0.030			*LIMD2*	0.041	*STAT4*	0.035	*RAB3B*	0.014
*USP13*	0.030			*BCKDK*	0.044	*DAP3*	0.035	*PTP4A2*	0.016
*SEPHS1*	0.030			*TRAPPC6A*	0.044	*TCERG1*	0.035	*NAP1L5*	0.016
*NRXN3*	0.035					*TATDN3*	0.035	*AKAP5*	0.017
*PAK3*	0.038					*MTF2*	0.035	*NXPH1*	0.023
*IGSF3*	0.039					*SLC20A1*	0.038	*PLXNC1*	0.023
*TSPYL4*	0.039					*ZNF652*	0.041	*PCDHGC5*	0.023
*SKP1*	0.039					*SLC4A1AP*	0.043	*NDFIP1*	0.023
*ZDHHC23*	0.042					*BTF3L4*	0.043	*ATP9B*	0.023
*GRM7*	0.042					*FBXL2*	0.043	*C11orf87*	0.023
*C2orf88*	0.042					*PCDH8*	0.043	*CAMK4*	0.024
*CKAP4*	0.046					*GULP1*	0.043	*COPS7A*	0.026
*CBX4*	0.049					*MFN1*	0.043	*PGAP1*	0.030
						*GRHL1*	0.043	*PPP1R14C*	0.030
						*FOXJ1*	0.043	*LRFN2*	0.036
						*KIF1C*	0.043	*STMN1*	0.048
						*CHD7*	0.043		
						*NRIP3*	0.047		
						*RHEB*	0.049		
						*MBTPS2*	0.049		
						*SEPT8*	0.049		

a Adjusted *p* value using FDR

Abbreviations: AD, Alzheimer’s dementia; FDR, false discovery rate; miRNAs, microRNAs; NCI, no cognitive impairment.

**Table 3:** KEGG pathway analysis of target genes of five AD-associated hub miRNAs (miR-129–5p, miR-1260, miR-200a, miR-433, and miR-221)

KEGG pathway	Number of target genes belonging to the pathway	Fold enrichment	*P* value ^a^
Axon guidance	31	2.931	<0.001
ErbB signaling pathway	20	4.048	<0.001
MAPK signaling pathway	35	1.994	0.012
GABAergic synapse	16	3.093	0.014
Autophagy - animal	21	2.562	0.015
AMPK signaling pathway	19	2.702	0.017
mTOR signaling pathway	22	2.426	0.020
Glutamatergic synapse	18	2.693	0.032

a Adjusted *p* value from Bonferroni correction

Abbreviations: AD, Alzheimer’s dementia; AMPK, 5’ adenosine monophosphate-activated protein kinase; ErbB, erythroblastic leukemia viral oncogene homolog; GABAergic, gamma-aminobutyric acid-ergic; KEGG, Kyoto Encyclopedia of Genes and Genomes; MAPK, mitogen-activated protein kinase; miRNAs, microRNAs; mTOR, mammalian target of rapamycin.

**Table 4:** List of miRNAs with kME > 0.7 for the CM2 module in the replication dataset

miRNA	kME	*P*value ^a^
**miR-744**	0.946	1.02 × 10^−6^
**let-7e**	0.892	4.60 × 10^−5^
**miR-485-3p**	0.887	4.60 × 10^−5^
**miR-769-3p**	0.883	4.60 × 10^−5^
**miR-491-5p**	0.881	4.60 × 10^−5^
**miR-331-3p**	0.863	9.46 × 10^−5^
**miR-107**	0.850	1.51 × 10^−4^
**miR-129-5p**	0.833	2.58 × 10^−4^
**miR-221**	0.827	2.77 × 10^−4^
**miR-129-3p**	0.825	2.77 × 10^−4^
**miR-770-5p**	0.823	2.77 × 10^−4^
**miR-708**	0.807	4.55 × 10^−4^
**miR-487a**	0.753	1.99 × 10^−3^
**miR-125a-5p**	0.716	4.42 × 10^−3^

a Adjusted *p* value using FDR

kME and *p* values represent the correlation and significance levels, respectively, between the miRNA expression levels and module eigengene of the consensus CM2 module. The miRNAs with kME > 0.7 in the replication dataset (GEO) are listed for the CM2 module.

Abbreviations: CM, consensus module; FDR, false discovery rate; GEO, Gene Expression Omnibus; ME, module eigengene; miRNAs, microRNAs.

**Table 5:** Mean AUC and standard deviation of machine learning models using penalized logistic regression

Model	Training features	Mean AUC	Standard deviation of AUC
**1**	Age + Sex + *APOE* ε4 carrier status	0.807	0.103
**2**	Five AD-associated hub miRNAs	0.740	0.068
**3**	Age + Sex + *APOE* ε4 carrier status + Five AD-associated hub miRNAs	0.870	0.061
**4**	All 309 miRNAs	0.815	0.052
**5**	Age + Sex + *APOE* ε4 carrier status + All 309 miRNAs	0.867	0.057

10-fold cross validation was used to investigate and compare the classification performance of five different machine learning models for differentiating AD from NCI. The machine learning model, mean AUC, and standard deviation of the AUC are presented.

Abbreviations: AD, Alzheimer’s dementia; *APOE*, apolipoprotein E; AUC, area under the curve; miRNAs, microRNAs, NCI, no cognitive impairment.

## Data Availability

All data sources mentioned in the study are publically available summary level information. The ROS/MAP cohort and GEO data will be freely available at the AMP-AD database (www.synapse.org) and the National Center for Biotechnology Information as accession numbers GSE157239 (http://www.ncbi.nlm.nih.gov/projects/geo/), respectively.

## Abbreviations

Aβ: amyloid-β
AD: Alzheimer’s dementia
AMP-AD: Accelerating Medicines Partnership for Alzheimer’s Disease
AMPK: 5′ adenosine monophosphate-activated protein kinase
APOE: apolipoprotein E
APP: amyloid precursor protein
AUC: area under the receiver operating characteristic curve
BP: biological processes
CERAD: Consortium to Establish a Registry for Alzheimer’s Disease
D: density
DAVID: Database for Annotation, Visualization and Integrated Discovery
DLPFC: dorsolateral prefrontal cortex
ErbB: erythroblastic leukemia viral oncogene homolog
FDR: false discovery rate
GABAergic: gamma-aminobutyric acid-ergic
GEO: Gene Expression Omnibus
GO: Gene ontology
HMGB1: high-mobility group box 1
KEGG: Kyoto Encyclopedia of Genes and Genomes
MAPK: mitogen-activated protein kinase
ME: module eigengene
miRNAs: microRNAs
mRNAs: messenger RNAs
mTOR: mammalian target of rapamycin
NCI: no cognitive impairment
NFTs: intracellular neurofibrillary tangles
NPs: neuritic plaques
qRT-PCR: real-time reverse transcription PCR ()
ROC: receiver operating characteristic
ROS/MAP: Religious Orders Study and Rush Memory and Aging Project
TOM: Topological Overlap Matrix
WGCNA: weighted gene co-expression network analysis
YAP1: yes-associated protein 1
3′-UTR: 3′-untranslated regions
